# Low Release Study of Cefotaxime by Functionalized Mesoporous Silica Nanomaterials

**DOI:** 10.3390/gels8110711

**Published:** 2022-11-03

**Authors:** Dan Eduard Mihaiescu, Daniela Istrati, Alina Moroșan, Maria Stanca, Bogdan Purcăreanu, Rodica Cristescu, Bogdan Ștefan Vasile, Roxana Doina Trușca

**Affiliations:** 1 Department of Organic Chemistry “Costin Nenitescu”, Faculty of Chemical Engineering and Biotechnologies, University Politehnica of Bucharest, 011061 Bucharest, Romania; 2 Leather and Footwear Research Institute, 031215 Bucharest, Romania; 3 Faculty of Chemical Engineering and Biotechnologies, Department of Science and Engineering of Oxide Materials and Nanomaterials, University Politehnica of Bucharest, 011061 Bucharest, Romania; 4 BIOTEHNOS S.A., Gorunului Rue, No. 3-5, 075100 Otopeni, Romania; 5 Lasers Department, National Institute for Lasers, Plasma and Radiation Physics, 077125 Magurele, Romania

**Keywords:** MCM-41, sol–gel, low release, cefotaxime, mesoporous material

## Abstract

As a third-generation β-lactam antibiotic, cefotaxime shows a broad-spectrum with Gram-positive and Gram-negative bacteria activity and is included in WHO’s essential drug list. In order to obtain new materials with sustained release properties, the present research focuses on the study of cefotaxime absorption and desorption from different functionalized mesoporous silica supports. The MCM-41-type nanostructured mesoporous silica support was synthesized by sol–gel technique using a tetraethyl orthosilicate (TEOS) route and cetyltrimethylammonium bromide (CTAB) as a surfactant, at room temperature and normal pressure. The obtained mesoporous material (MCM-41 class) was characterized through nuclear magnetic resonance (NMR), scanning electron microscopy (SEM), high-resolution transmission electron microscopy (HR-TEM), N_2_ absorption–desorption (BET) and Fourier transform infrared spectroscopy (FT-IR), proving a good micro-structured homogeneity (SEM images), a high surface area (BET, 1029 m^2^/g) correlated with high silanolic activity (Q^3^/Q^4^ peak ratio from ^29^Si MAS-NMR), and an expected uniform hexagonal structure (2–3 nm, HRTEM). In order to non-destructively link the antibiotic compound on the solid phase, MCM-41 was further functionalized in two steps: with aminopropyl trimethoxysilane (APTMS) and glutaraldehyde (GA). Three cefotaxime-loaded materials were comparatively studied for low release capacity: the reference material with adsorbed cefotaxime on MCM-41, MCM-41/APS (aminopropyl silyl surface functionalization) adsorbed cefotaxime material, and APTMS–GA bounded MCM-41—cefotaxime material. The slow-release profiles were obtained by using an on-flow modified HPLC system. A significant improved release capacity was identified in the case of MCM-41/APS/GA—cefotaxime due to the covalent surface grafting of the biological active compound, recommending this class of materials as an effective carrier of bioactive compounds in wound dressing, anti-biofilm coatings, advanced drugs, and other related applications.

## 1. Introduction

The discovery of MCM-41-type mesoporous silica could be consider a milestone in the field of silica-based materials due to the uniform hexagonal pore structure, the high surface area [1], chemical, thermal stability, biocompatibility, and high loading capacity [2,3]. The abundance of silanol groups on the surface of mesoporous silica nanoparticles (MSNs) allows for enhanced flexibility for surface modification through functionalization; thus, widening their range of application. MSNs are used in various applications such as therapeutic/health, drug delivery, agricultural fields, food industries, optoelectronic sensing [4,5], catalysis [6,7,8,9], gas separation [10,11], and lanthanides recovery from waste water [12,13]. In addition, organic/inorganic micro/nanocomposites using oxidic-based microporous structures and different strategies of surfaces functionalization are used as innovative solutions for advanced waste water treatments and pathogen inactivation approaches [14,15]. An important application of mesoporous silica consists of its usage as a carrier for biologically active compounds, such as gemcitabine [16] and quercetin [17], both used in cancer treatment. The main advantages of using mesoporous silica nanocomposites in biomedical research and application fields include their good biocompatibility, increased bio-availability of the target compounds for the tissue of interest (as example tumor tissue), sustained or controlled release of the bioactive compounds, and reduced toxicity of these compounds in healthy tissues [18,19].

V. Pardhi and collaborators improved the dissolution rate of niclosamide in the human body by using mesoporous silica as a delivery system and studied the influence of mesoporous silica morphology on the dissolution rate [20]. The study demonstrated that niclosamide loaded on mesoporous silica has a higher dissolution rate than the drug as such and silica morphology also influences the dissolution rate, this being lower for non-porous silica and higher for non-ordered mesoporous silica.

V. Nairi et al. studied the adsorption and release of ampicillin from mesoporous silica matrices, which differ from each other either by pore size or chemical properties due to the surface functionalization. The compared materials differ in pore size equivalent to electric charge (SBA-15 and MCM-41) and different surface electric charge (SBA-15 and SBA-15-NH_2_). Both the adsorption and release of ampicillin are influenced by the surface electric charge more than the pore size. The surface negative charge of the two non-functionalized matrices favors rapid release at pH 7.4, being slightly higher for MCM-41. As the MCM-41 particles have smaller dimensions than the SBA-15 ones, a higher amount of drug was adsorbed. In the case of the SBA-15-NH_2_ matrix, the electrostatic interactions favor an antibiotic sustained release that is slower than that of the nonfunctionalized material [21]. The biological responses could be enhanced by modifying the surface of mesoporous silica, such as the surface functionalization by attaching specific functional groups or molecules. The most common methods of functionalization are co-condensation and post-grafting. The selected approach influences both the physicochemical interaction and surface chemistry modification. The co-condensation or direct synthesis functionalization method can be performed in a single reaction vessel; and thus, has a reduced number of steps and synthesis time, leading to a more homogeneous distribution of functional groups on the surface. On the other hand, the post-grafting method uses simple and mild conditions, and involves the subsequent surface modification by direct grafting or secondary grafting. Since only molecules that are small enough can diffuse into the mesoporous matrix, this functionalization method is limited by both the pore size and architecture [22,23].

The special structure of mesoporous silica offers an efficient protection of biomolecules susceptible to metabolic transformations in the gastrointestinal tract after oral administration [24]. Silicon is a structural component of connective tissues, being essential for bones [25] and skin [26]. Acute and chronic toxicity studies have shown that the mesoporous silica does not have adverse effects for oral administration due to its low water solubility; however, the long-term pulmonary exposure to amorphous or crystalline silica (solid dispersions in the gas phase) has been associated to serious respiratory disorders [27,28].

Cefotaxime is a third-generation cephalosporin antibiotic that when it is intravenously or intramuscularly administered, becomes active against Gram-positive and Gram-negative bacteria, except pseudomonas. It is used to treat meningitis, lower respiratory tract infections, urinary tract, inflammatory pelvic diseases, skin infections, and gonorrhea [29,30]. Cefotaxime has the ability to inhibit bacterial cell wall synthesis compared to penicillin by blocking the transpeptidation step in peptidoglycan biosynthesis [31]. The cell envelope of Gram-positive and Gram-negative bacteria consist of a plasma membrane and cell wall. The difference between those two is that the Gram-negative bacteria poses an additional outer impermeable membrane to large molecules. The cell walls are similar (a single layer of peptidoglycan); however, they differ in thickness: 20–80 nm for Gram-positive bacteria and less than 10 nm for Gram-negative bacteria [32,33]. A desorption study of cefotaxime from MCM-41 and MCM-41/APS materials proves significant advantages of the amino groups’ (-NH_2_) surface functionalization [34].

The present research focuses on the study of the absorption/desorption of cefotaxime from functionalized mesoporous silica support, specifically focusing on the significant release difference between the non-destructively linked cefotaxime and physically adsorbed compound on the solid phase. Furthermore, a different low-release experimental system was proposed, using a modified HPLC system (avoiding sampling and HPLC injection) that is able to provide on-flow analysis (more than 4000 sampling points for a single experiment).

## 2. Results and Discussion

The synthesis of cefotaxime-bonded MCM-41/APS/GA composites involved several reaction steps, followed by advanced investigations of the intermediate and final mate-rials: MCM-41 synthesis by room temperature/pressure sol–gel method, APTMS surface functionalization in order to provide a -NH_2_ linking center for the glutaraldehyde surface grafting step, followed by the final cefotaxime (non-destructive) linking to the free thiazolyl amino group. Further desorption studies were performed in order to provide the release profiles of three materials: MCM-41—cefotaxime (3:10 (*w/w*)), MCM-41/APS—cefotaxime (3:10 (*w/w*)) loaded materials (physical adsorption), and covalent-linked cefotaxime MCM-41/APS/GA (3:10 (*w/w*)). In order to obtain an effective concentration of the drug in the desorption environment, the 3:10 (*w/w*) loading level of cefotaxime was established by the previous bioactive compound loading studies [35], which provided a significant out-of-pore compound concentration (the quick release of out-of-pore cefotaxime provides a fast response in the first stage of treatment), followed by a sustained release (in-pore desorption, in order to sustain the already established cefotaxime concentration). The cefotaxime GA cross-linked composite provides a significantly lower drug release due to the involvement of the secondary aldiminic bound cleavage from the solid support. This behavior would be a significant advantage in wound dressing, advanced drugs design, and antibiofilm coatings applications because the pH drop from bacterial activity will be followed by the desorption enhancement due to an accelerated aldiminic-bound cleavage.

**Figure 1:**
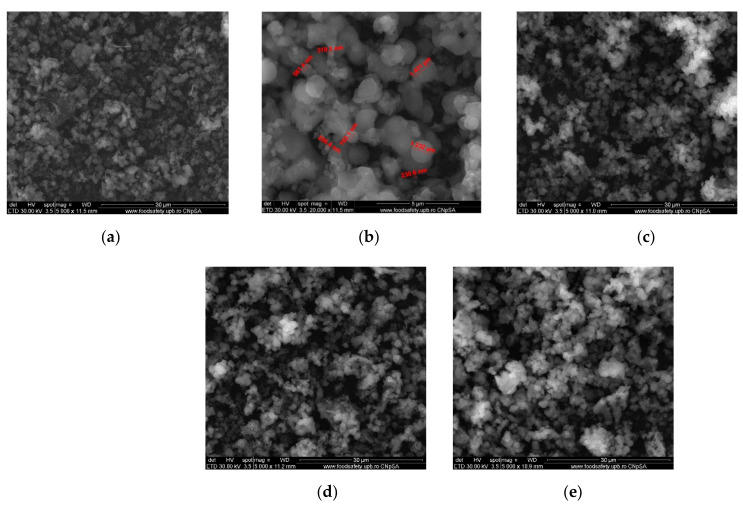
SEM images for initial MCM-41 material (**a**), MCM-41 with particle size details (**b**), MCM-41/APS (**c**), MCM-41/APS/GA (**d**) and MCM-41/APS/GA-cefotaxime (**e**).

SEM images (Figure 1) of MCM-41 (a), MCM-41 with particle size details (b), MCM-41/APS (c), MCM-41/APS/GA (d), and cefotaxime-linked MCM-41/APS/GA (e) materials exhibit a relative uniform microgranular structure both in shape and particle size. APTMS surface functionalization, the glutaraldehyde surface-grafting step, and the final cefotaxime-loading process do not affect the morphology of the composites; in terms of both shape and particle size, a slight particle aggregation can be observed (e) for the loaded material, as expected from the out-of-pore cefotaxime loading. The microstructured spherical particle distribution was the subject of several MCM-41 synthesis optimization steps, avoiding as possible the low-diameter nanoparticles (almost pharmaceutical release applications avoiding nanometer scale-range particles due to the significant tissue penetration).

**Figure 2:**
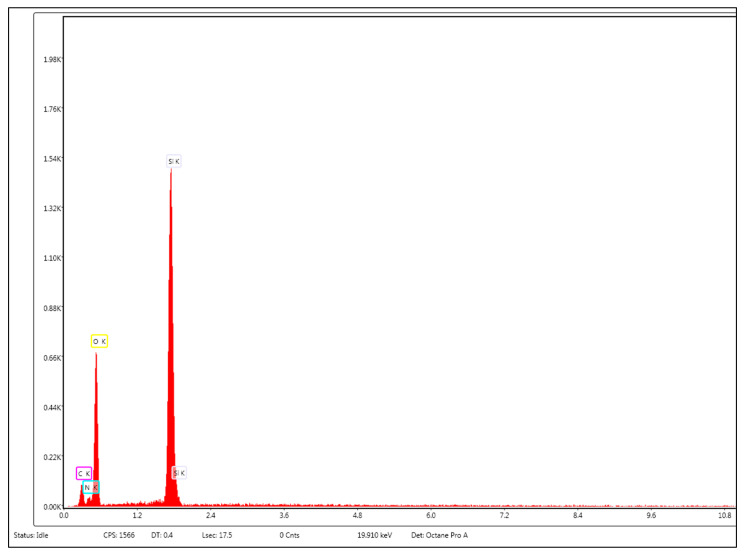
The EDS spectrum for MCM-41/APS.

Functionalization of MCM-41 with APTMS is demonstrated by the appearance of the peak corresponding to nitrogen; in addition, the peaks corresponding to silicon and oxygen are only revealed by energy dispersive spectroscopy (EDS) (Figure 2).

**Figure 3:**
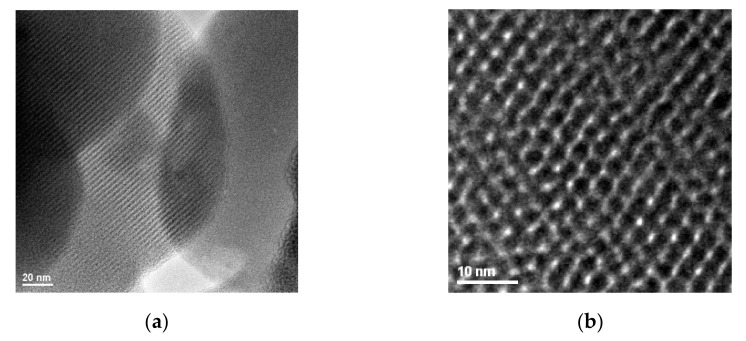
Representative HR-TEM images of non-functionalized MCM-41 material for 20 nm (**a**) and MCM-41/APS for 10 nm (**b**) scale bars.

Figure 3 exhibits HR-TEM images of non-functionalized MCM-41 material (because of the high electron energy, surface grafted materials are less suitable for this analysis). The HR-TEM images confirm the MCM-41 granular mesoporous silica morphology (200–1500 nm particle size) and its mesoporous hexagonal structure. For the MCM-41 synthesis operated at normal pressure and temperature, the obtained structure shows a good hexagonal ordering degree and an average pore size of about 2–3 nm, proved also by the high surface area.

**Figure 4:**
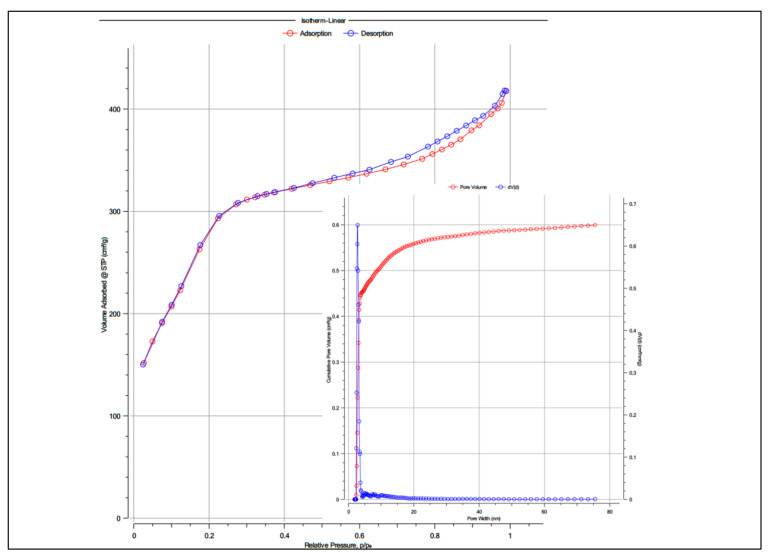
N_2_ adsorption/desorption isotherm and pore size distribution for MCM-41.

According to IUPAC, the N_2_ adsorption/desorption isotherm is type IV, specific to MCM-41 type materials (Figure 4). This feature highlights the characteristics of the MCM-41 material: the specific surface area of 1029.06 m^2^ g^−1^ pore sizes of 2.41 nm and pore volume of 0.62 cm^3^ g^−1^, being characteristic of mesoporous materials, which also confirms our HR-TEM data. The pore size distribution (Figure 4) confirms the uniformity of the pore diameter.

**Figure 5:**
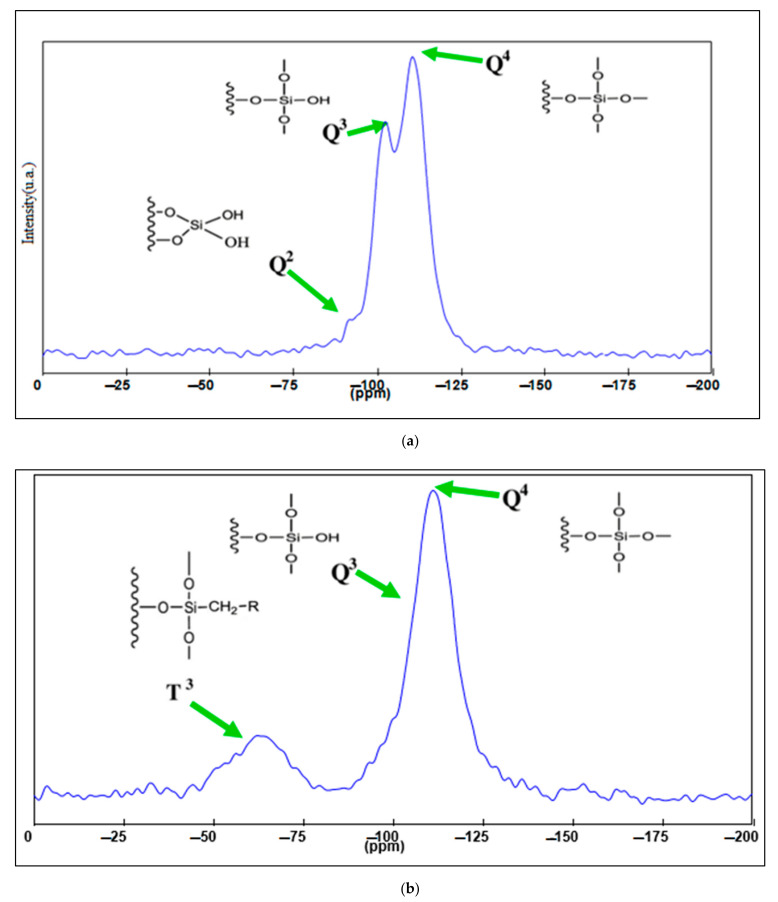
^29^Si MAS NMR spectrum (8 kHz rotation frequency) of non-functional mesoporous silica (**a**) and APS-grafted (**b**) materials.

^29^Si –NMR MAS analysis of the MCM-41 and MCM-41/APS materials highlight some unique structural aspects of the mesoporous silica silanolic surface, such as the presence of silicon atoms Q^4^, Q^3^, and Q^2^ (single and geminal silanolic groups). Figure 5a shows a small Q^2^ geminal and high Q^3^ bands, evidencing the presence of almost single silanolic groups on the silica surface. The APTMS functionalization provides effective binding of MCM-41 single silanolic groups (Figure 5b), Q^3^ band significant mitigation correlated with the presence of a T^3^ band, attesting to the existence of a new covalent silicon—carbon bound [36].

In order to correlate the specific vibrational information with the MAS-NMR results, further FT-IR and Raman investigations of the intermediate and final materials were performed.

**Figure 6:**
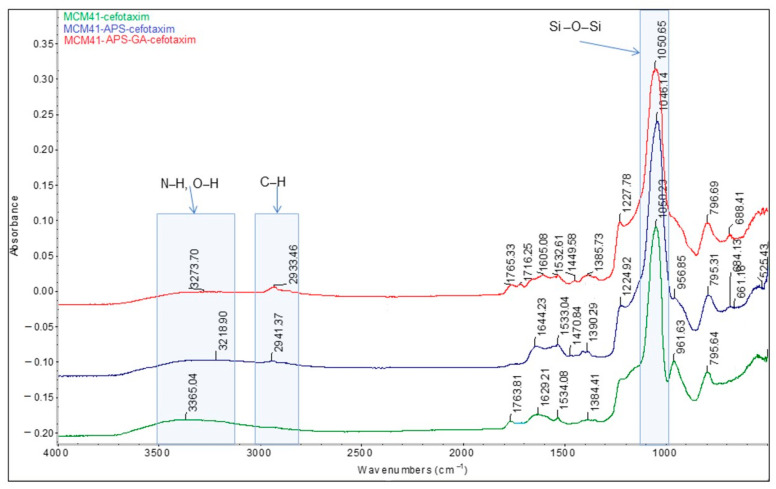
Comparative horizontal attenuated total reflectance (HATR)-FT-IR spectra of MCM-41/cefotaxime, MCM-41/APS—cefotaxime, and MCM-41/APS/GA—cefotaxime (the final 3 cefotaxime-loaded materials used for the desorption experiments).

The presence of the saturated chain of glutaraldehyde is confirmed by the absorption band at 2933 cm^−1^, with a significantly higher intensity compared to the MCM-41/APS band (2941 cm^−1^) correlated with the presence of the propyl chain (Figure 6).

**Figure 7:**
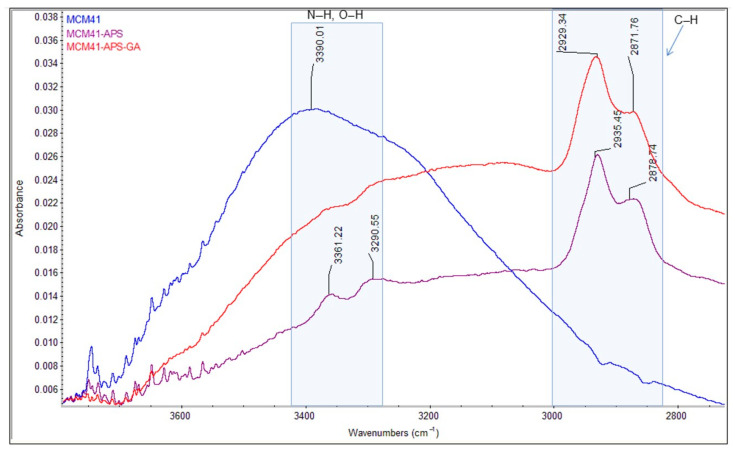
Comparative HATR-FT-IR spectra of MCM-41, MCM-41/APS, and MCM-41/APS/GA materials.

**Figure 8:**
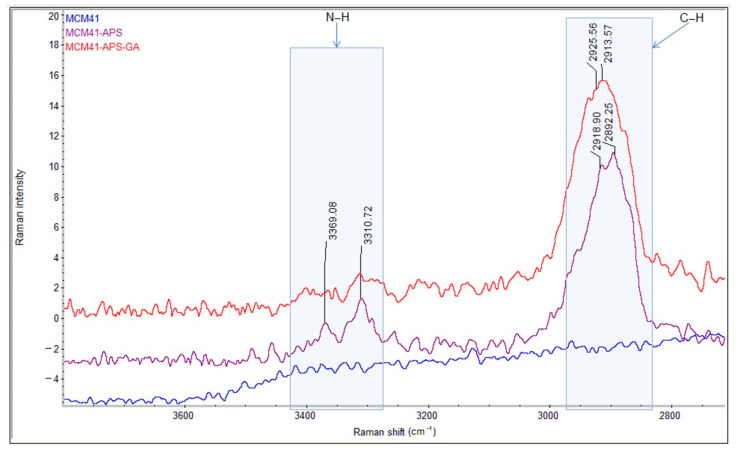
Comparative Raman spectra of MCM-41, MCM-41/APS, and MCM-41/APS/GA materials.

The comparative FT-IR and Raman spectra of MCM-41, MCM-41/APS, and MCM-41/APS/GA confirm the presence of the saturated chain of GA due to the presence of absorption bands centered at 2929 and 2871 cm^−1^, respectively, in Figure 7; and 2925 and 2913 cm^−1^, respectively, in Figure 8. The absorption bands centered at 2935 and 2878 cm^−1^, respectively, in Figure 7; and 2918 and 2892 cm^−1^, respectively, in Figure 8, confirm the presence of APS-saturated chains; while the -NH_2_ groups can be observed at 3361 and 3290 cm^−1^, respectively, in Figure 7; and 3369 and 3310 cm^−1^, respectively, in Figure 8 (correlated to the presence of the peak corresponding to nitrogen in the EDS spectrum Figure 2 and T^3^ peak from MAS-NMR Figure 5). Moreover, the intensity of the characteristic bands of -NH_2_ groups from the MCM-41/APS/GA significantly decreased in comparison to the mesoporous silica–amino groups.

**Figure 9:**
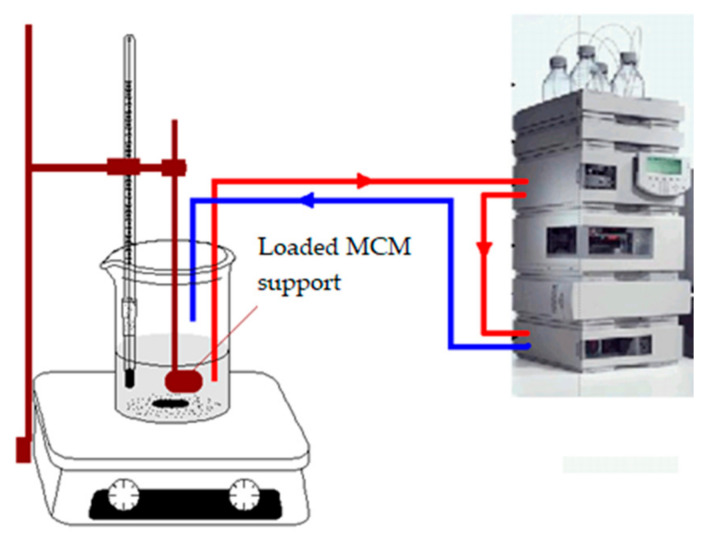
The experimental system (modified HPLC) used for the desorption profile acquisition.

In order to estimate the release particularities linked to the different interactions between the biologically active compound and the solid mesoporous support, the final step of this study was related to the comparative low-release capacity evaluation of the three loaded materials: MCM-41—cefotaxime, MCM-41/APS—cefotaxime, and MCM-41/APS/GA—cefotaxime. The experimental system (Figure 9) uses an Agilent 1200 series HPLC system with a manual injector, with several significant modifications: the solvent feed line of the upper mobile phase bottle was inserted in the release vessel, directly passing the liquid phase to the HPLC pump and further through the injection loop; the column was bypassed (the liquid flow from the pump was directly connected with the UV–Vis detector); and the detector exhaust line was redirected to the release vessel for the purpose of closing the whole on-flow loop. Together with the insertion of the desorption bag in the release solution, a formal blank manual injection was performed to start the data acquisition, the acquired “chromatogram” actually containing the desired desorption profile. For a proper decontamination of the whole system and cross-contamination mitigation, several washing steps were performed after each experiment, with different liquid phase pH values and baseline monitoring. For quantitative measurements, the calibration profiles were obtained and a mean value of 50 data points from each calibration profile was used for the linear regression calculations (the data points were extracted from the flat final region of the calibration profiles; the calibration samples were fully dissoluted, yielding a final flat profile of the desorption curve after 10 min of release).

In comparison to other on-flow or discontinuous sampling systems, our proposed desorption system provides significant advantages: high sampling rates (5–20 Hz range, feasible values for the proposed experiments are lower than 5 Hz due to the low absorbance changes compared to a normal HPLC analysis) and smooth shape of the desorption profile; low detection limits—correlated with the high HPLC detector performance; a low dead volume due to the HPLC lines; low volume of the UV–Vis detection flow cell; and long-term desorption capability (more than 48 h with a proper experiment setup).

All the desorption experiments were performed in ultrapure water (at 6.5 pH), using a similar experiment setup and acquisition conditions.

**Figure 10:**
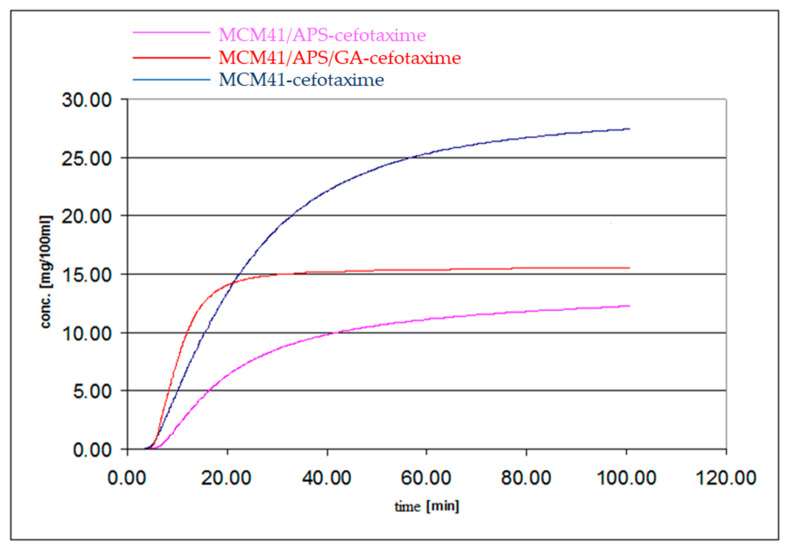
Desorption profiles of cefotaxime on the three support types.

A significant release difference between the non-destructively linked cefotaxime and physically adsorbed compound on the APS functionalized MCM-41 solid phase can be established from Figure 10. Significant release differences are observed in the two important regions of the desorption profiles: the first region involves a fast release of cefotaxime in the first 20–30 min, while the final region, with a significant slope difference, involves a low, sustained release of the compound. The GA covalent-linked cefotaxime composite shows an intermediate release concentration (between the other two materials) after 120 min; however, it shows a significant lower release trend. The higher cefotaxime release concentration, compared to the MCM-41/APS adsorbed compound one, could be explained by a lower in-pore surface access of cefotaxime after the glutaraldehyde link (in the final loading stage of composite synthesis), by a higher amount of the out-of-pore cefotaxime available for a fast release in the first 20–30 min. Following the final slope trend of the two profiles, there is expected to be a higher release concentration of the MCM-41/APS adsorbed cefotaxime at several hours of desorption time (overpassing the GA-bonded cefotaxime profile) due to the differences in the release mechanism (desorption and aldiminic bond cleavage, respectively).

The significant slope difference of the two profiles in the first 20 min of desorption can be explained by a lower interaction of the out-of-pore cefotaxime with the GA-grafted surface due to the hydrophobic saturated carbon chain of glutaraldehyde, yielding an increased release of the out-of-pore cefotaxime.

Because of the significant differences of the silanolic access of loaded cefotaxime, the non-functionalized MCM-41 material shows a significant release difference when compared to functionalized materials. In addition, due to the higher access of water at the polar silanolic surface, an enhanced release of cefotaxime is expected (because of the hydrogen bond interaction of cefotaxime with the silanolic groups, expected at -NH-, -NH_2_, and -OH sites; the water dissolution rate will be controlled by the water molecules access at the silanolic surface. For the APS-grafted surface, the water access at the silanolic groups is significantly reduced, providing a lower release compared to MCM-41. The APS/GA material exposes a significantly lower polarity surface to the water environment; and thus, a faster release of the out-of-pore absorbed cefotaxime is expected). Despite the significant differences, a similarity between the final slopes of the MCM-41 and MCM-41/APS materials must be noticed, proving a similar in-pore diffusion mechanism. Certainly, this result correlated with the significant slope difference of the GA linked-material, which should sustain the desorption mechanism differences.

## 3. Conclusions

The present work describes the synthesis, characterization, and comparative low re-lease study of a new mesoporous composite material, with covalent cefotaxime grafting on a MCM-41/propylamino/glutaraldehyde (MCM-41/APS/GA) solid phase.

The obtained materials were characterized by BET, FT-IR, RAMAN, SEM, HR-TEM, and NMR investigation methods. MCM-41 synthesis was conducted to micrometer range particles (avoiding low-diameter silica nanoparticles), with a 1029 m^2^g^−1^ specific surface area (BET), pore size of 2.41 nm, and pore volume of 0.62 cm^3^ g^−1^; and a high silanolic surface trough Q^3^ silanolic group peak from the ^29^Si MAS NMR spectrum. The further APS surface functionalization (performed by MCM-41—APTMS reaction) was proved by the presence of free -NH_2_ groups (EDS, IR, Raman), and GA surface grafting was obtained by the glutaraldehyde aldiminic link. The last stage involved MCM-41, MCM-41/APS, and MCM-41/APS/GA materials loading with cefotaxime (3:10 (*w/w*).

We proposed a low release experimental system, using a modified HPLC system (avoiding sampling and HPLC injection) that is able to provide on-flow analysis (more than 4000 sampling points for a single experiment). The three mesoporous materials: MCM-41—cefotaxime, MCM-41/APS—cefotaxime, and MCM41/APS/GA-cefotaxime were tested in desorption experiments in water. A significant release difference between the non-destructively linked cefotaxime, physically adsorbed compound on the APS functionalized, MCM-41 solid phase was established. Moreover, significant release differences were observed in the two important regions of the desorption profiles: the first region involving a fast release of cefotaxime in the first 20–30 min and the final region, with a significant slope difference involving a low, sustained release of the compound. The GA covalent-linked cefotaxime composite shows an intermediate release concentration (between the other two materials) after 120 min; however, it shows a significant lower release trend. The low release capacity of the mesoporous glutaraldehyde-linked cefotaxime composite would be a significant advantage in wound dressing applications, advanced drugs design, antibiofilm coatings, and other related applications.

## 4. Materials and Methods

### 4.1. Reagents and Equipment

Cefotaxime hydrochloride, tetraethylorthosilicate (TEOS), glutaraldehyde (GA), aminopropyl trimethoxysilane (APTMS), methanol, ethanol, acetonitrile (ACN), and ammonium hydroxide solution 25% were purchased from Sigma-Aldrich and were used without further purification. Cetyltrimethylammonium bromide (CTAB) was purchased from Fluka and used as such. All the chemicals were of analytical purity and used as received.

Scanning electron microscope (SEM) QUANTA INSPECT F (Thermo Fisher—formerly FEI, Eindhoven, The Netherlands) field emission gun of resolution 1.2 nm was used to investigate the sample surface morphology, using energy dispersive X-ray (EDX) with the resolution to MnKα 133 eV.

High-resolution electron transmission microscope (HR-TEM) were performed on a Tecnai G2 F30 S-TWIN equipped with energy dispersive spectroscopy (EDS) as well as a selected area electron diffraction detector (SAED) purchased from Thermo Fisher—formerly FEI (Hillsboro, OR, USA). The microscope was operated in transmission mode at 300 kV; the HR-TEM point resolution was 2 Å and line resolution was 1 Å.

Fourier transform infrared (FT-IR) spectra were recorded using a Nicolet iS50FT-IR (Thermo Nicolet, Massachusetts, USA) spectrometer equipped with a DTGS detector and Raman accessory. The measurements were carried out in the range of 4000–400 cm^−1^, using the resolution 4 cm^−1^ and 100 scans per spectrum. All the spectra were recorded using horizontal attenuated total reflectance (HATR) with diamond crystal. FT-Raman spectra were collected using the same device, using an InGaAs detector, a CaF_2_ beamsplitter, 100 scans per spectrum, and the excitation laser power at 0.50 W.

The release profiles were recorded using a modified HPLC system, as previously presented [35].

Brunauer–Emmett–Teller (BET) analysis. The nitrogen adsorption/desorption isotherms were registered at 77 K in the relative pressure range p/po = 0.005–1.0, by a NOVA 800 Gas Sorption Analyzer (Anton Paar QuantaTec, Inc., Boyton Beach, FL, USA). Data processing was performed using Kaomi software. Prior to adsorption measurements, the samples were degassed up to 180 °C under vacuum for 4 h. The standard Brunauer–Emmett–Teller (BET) equation was used to determine the specific surface area. The gas volume absorbed at a relative pressure p/po~1 allowed the estimation of the total pore volume. The Barrett–Joyner–Halenda (BJH) model was used to obtain the pore size distribution and mesopore volume from the desorption branch of the isotherm.

NMR was performed using a Bruker Avance III spectrometer (Bruker, MA, USA), with a 14.1 Tesla magnet (600 MHz proton resonance frequency). The samples were analyzed in solid state (^29^Si) using a 3.2 mm diameter rotor and a rotational frequency of 8 kHz. For ^29^Si nuclei, the NMR MAS “one pulse” technique was used.

### 4.2. Synthesis

The method of mesoporous silica synthesis is similar to that of previously described work [35]. Further, the mesoporous MCM-41 material was functionalized with propyl-amino groups by reaction with APTMS in acetonitrile.

#### Pressure Vessel Functionalization

Mesoporous silica (100 mg) was mixed with APTMS–ACN solution (1:4). The reaction mixture was placed in a pressure-tight reaction vessel and kept for 12 h at 85 °C. After cooling, the mixture was separated by centrifugation, washed with ultrapure water (at least 5×), and dried at 105 °C for 7 h.

In addition, a second functionalization method (microwave assisted) was attempted in order to improve the APS surface grafting; however, the results obtained sustained an in-pore polycondensation of the silane with a large surface area loss. This surface functionalization method could still be a good alternative to pressured vessel synthesis, using low microwave (MW) exposure times and reaction conditions optimization.

Further grafting of glutaraldehyde on the amino functionalized silica surface was performed as follows: the amino functionalized material (100 mg) was dispersed in ultrapure water (2 mL) and mixed with 20% solution of glutaraldehyde (2 mL). The obtained mixture was kept for 5 h at 55 °C. After cooling, the solid was separated by centrifugation and washed (at least 5×) times with ultrapure water. The final material was dried for 7 h at 55 °C.

Cefotaxime loading was performed as follows: the functionalized amino–glutaraldehyde material (100 mg) was mixed with cefotaxime (30 mg) in 10 mL methanol and subsequently, dried under vacuum in a rotary evaporator at 30 °C. The solvent evaporation process provides a cefotaxime concentration gradient, respectively, a high pore loading of the final product, and an out-of-pore distribution of the cefotaxime excess. A final drying stage was performed for 12 h at 55 °C. This method assures no bioactive compound loss in the loading stage (compared to the liquid phase loading methods that involve a solid–liquid partition).

## Data Availability

Available on demand.

## References

[1] Singh B., Na J., Konarova M., Wakihara T., Yamauchi Y., Salomon C., Gawande M.B. (2020). Functional Mesoporous Silica Nanomaterials for Catalysis and Environmental Applications. Bull. Chem. Soc. Jpn..

[2] Gustafsson H., Isaksson S., Altskär A., Holmberg K. (2016). Mesoporous silica nanoparticles with controllable morphology prepared from oil-in-water emulsions. J. Colloid Interface Sci..

[3] Popova T., Voycheva C., Tzankov B. (2020). Study on the influence of technological factors on drug loading of poorly water-soluble drug on MCM-41 mesoporous carrier. Pharmacia.

[4] Nayl A.A., Abd-Elhamid A.I., Aly A.A., Bräse S. (2022). Recent progress in the applications of silica-based nanoparticles. RSC Adv..

[5] Martínez-Carmona M., Gun’ko Y.K., Vallet-Regí M. (2018). Mesoporous Silica Materials as Drug Delivery: “The Nightmare” of Bacterial Infection. Pharmaceutics.

[6] Jeenpadiphat S., Björk E.M., Odén M., Tungasmita D.N. (2015). Propylsulfonic acid functionalized mesoporous silica catalysts for esterification of fatty acids. J. Mol. Catal. A Chem..

[7] More P.M., Umbarkar S.B., Dongare M.K. (2016). Template-free sol–gel synthesis of high surface area mesoporous silica based catalysts for esterification of di-carboxylic acids. Comptes Rendus Chim..

[8] Sánchez-Bastardo N., Alonso E. (2017). Maximization of monomeric C5 sugars from wheat bran by using mesoporous ordered silica catalysts. Bioresour. Technol..

[9] Nejati-Shendi M., Tebyanian H., Zare R., Ayoubi-Chianeh M., Roshani K., Kassaee M.Z., Rashidiani J. (2020). Hollow Mesoporous Silica Sphere (HMSS) as a RecyclableNano-catalyst in an Efficient One-Pot MulticomponentSynthesis of 2-Amino-3-Cyano-4H-Pyran Derivatives. Biointerface Res. Appl. Chem..

[10] Waheed N., Mushtaq A., Tabassum S., Gilani M.A., Ilyas A., Ashraf F., Jamal Y., Bilad M.R., Khan A.U., Khan A.L. (2016). Mixed matrix membranes based on polysulfone and rice husk extracted silica for CO2 separation. Sep. Purif. Technol..

[11] Zornoza B., Téllez C., Coronas J. (2011). Mixed matrix membranes comprising glassy polymers and dispersed mesoporous silica spheres for gas separation. J. Membr. Sci..

[12] Ramasamy D.L., Khan S., Repo E., Sillanpää M. (2017). Synthesis of mesoporous and microporous amine and non-amine functionalized silica gels for the application of rare earth elements (REE) recovery from the waste water-understanding the role of pH, temperature, calcination and mechanism in Light REE and Heavy REE separation. J. Chem. Eng..

[13] Ramasamy D.L., Repo E., Srivastava V., Sillanpää M. (2017). Chemically immobilized and physically adsorbed PAN/acetylacetone modified mesoporous silica for the recovery of rare earth elements from the waste water-comparative and optimization study. Water Res..

[14] Karki S., Ingole P.G. (2022). Development of polymer-based new high performance thin-film nanocomposite nanofiltration membranes by vapor phase interfacial polymerization for the removal of heavy metal ions. Chem. Eng. J..

[15] He J., Zeng X., Lan S., Lo I.M.C. (2019). Reusable magnetic Ag/Fe, N-TiO_2_/Fe_3_O_4_@SiO_2_ composite for simultaneous photocatalytic disinfection of E. coli and degradation of bisphenol A in sewage under visible light. Chemosphere.

[16] Dai J.-T., Zhang Y., Li H.-C., Deng Y.-H., Elzatahry A.A., Alghamdi A., Fu D.-L., Jiang Y.-J., Zhao D.-Y. (2017). Enhancement of gemcitabine against pancreatic cancer by loading in mesoporous silica vesicles. Chin. Chem. Lett..

[17] Sarkar A., Ghosh S., Chowdhury S., Pandey B., Sil P.C. (2016). Targeted delivery of quercetin loaded mesoporous silica nanoparticles to the breast cancer cells. Biochim. Biophys. Acta-Gen. Subj..

[18] Niculescu V.-C. (2020). Mesoporous Silica Nanoparticles for Bio-Applications. Front. Mater..

[19] Vadia N., Rajput S. (2019). Importance & Applications of Nanotechnology. Applications of Mesoporous Material for Drug Delivery.

[20] Pardhi V., Chavan R.B., Thipparaboina R., Thatikonda S., Naidu V.G.M., Shastri N.R. (2017). Preparation, characterization, and cytotoxicity studies of niclosamide loaded mesoporous drug delivery systems. Int. J. Pharm..

[21] Nairi V., Medda L., Monduzzi M., Salis A. (2017). Adsorption and release of ampicillin antibiotic from ordered mesoporous silica. J. Colloid Interface Sci..

[22] Chircov C., Spoială A., Păun C., Crăciun L., Ficai D., Ficai A., Andronescu E., Turculeƫ Ș.C. (2020). Mesoporous Silica Platforms with Potential Applications in Release and Adsorption of Active Agents. Molecules.

[23] Sartori B., Amenitsch H., Marmiroli B. (2021). Functionalized Mesoporous Thin Films for Biotechnology. Micromachines.

[24] Diab R., Canilho N., Pavel I.A., Haffner F.B., Girardon M., Pasc A. (2017). Silica-based systems for oral delivery of drugs, macromolecules and cells. Adv. Colloid Interface Sci..

[25] Jugdaohsingh R. (2007). Silicon and bone health. J. Nutr. Health Aging.

[26] de Araújo L.A., Addor F., Campos P.M.B.G.M. (2016). Use of silicon for skin and hair care: An approach of chemical forms available and efficacy. An. Bras. Dermatol..

[27] Bhattacharjee P., Paul S., Bhattacharjee P. (2016). Risk of occupational exposure to asbestos, silicon and arsenic on pulmonary disorders: Understanding the genetic-epigenetic interplay and future prospects. Environ. Res..

[28] Khan A.H., Bienia O.B. (2021). Identifying Potential Inhalation Risks Associated with Exposure to Different Forms of Silica at the Nanomolecular Level as it Relates to Antimicrobial Agents. Adv. Biotechnol. Microbiol..

[29] Beckett W., Enna S.J., Bylund D.B. (2008). Airways Disease. xPharm: The Comprehensive Pharmacology Reference.

[30] Hathout R.M., Abdelhamid S.G., El-Housseiny G.S., Metwally A.A. (2020). Comparing cefotaxime and ceftriaxone in combating meningitis through nose-to-brain delivery using bio/chemoinformatics tools. Sci. Rep..

[31] LeFrock J.L., Prince R.A., Leff R.D. (1982). Mechanism of action, antimicrobial activity, pharmacology, adverse effects, and clinical efficacy of cefotaxime. Pharmacotherapy.

[32] Varghese M., Balachandran M. (2021). Antibacterial efficiency of carbon dots against Gram-positive and Gram-negative bacteria: A review. J. Environ. Chem. Eng..

[33] He J., Zheng Z., Lo I.M.C. (2021). Different responses of gram-negative and gram-positive bacteria to photocatalytic disinfection using solar-light-driven magnetic TiO_2_-based material, and disinfection of real sewage. Water Res..

[34] Maria G., Stoica A.-I., Luta I., Stirbet D., Radu G.L. (2012). Cephalosporin release from functionalized MCM-41 supports interpreted by various models. Microporous Mesoporous Mater..

[35] Istrati D., Mihaiescu D.E., Gudovan D., Gudovan I.A., Traistaru V., Marton A. (2015). Controlled release profiles of Cefepime from MCM-41-NH2 materials. Rev. Roum. Chim..

[36] Protsak I.S., Morozov Y.M., Dong W., Le Z., Zhang D., Henderson M. (2019). A ^29^Si, ^1^H, and ^13^C Solid-State NMR Study on the Surface Species of Various Depolymerized Organosiloxanes at Silica Surface. Nanoscale Res. Lett..

